# Electron-Beam Irradiated Recombinant Human Collagen-Phosphorylcholine Corneal Implants Retain Pro-Regeneration Capacity

**DOI:** 10.3389/fbioe.2022.883977

**Published:** 2022-06-13

**Authors:** Fiona C. Simpson, Mohammed Mirazul Islam, Oleksiy Buznyk, Elle Edin, Marc Groleau, Monika Kozak-Ljunggren, Federica M. Magrelli, Dina B. AbuSamra, Pablo Argüeso, James Chodosh, Aneta Liszka, Per Fagerholm, May Griffith

**Affiliations:** ^1^ Department of Clinical and Experimental Medicine, Linköping University, Linköping, Sweden; ^2^ Maisonneuve-Rosemont Hospital Research Centre, Montréal, QC, Canada; ^3^ Department of Ophthalmology, Institute of Biomedical Engineering, Université de Montréal, Montréal, QC, Canada; ^4^ Centre de Recherche—Centre Hospitalier de l’Université de Montréal, Montréal, QC, Canada; ^5^ Department of Ophthalmology, Massachusetts Eye and Ear and Schepens Eye Research Institute, Harvard Medical School, Boston, MA, United States; ^6^ Filatov Institute of Eye Diseases and Tissue Therapy of the NAMS of Ukraine, Odessa, Ukraine; ^7^ Department of Polymer Chemistry, Uppsala University, Uppsala, Sweden; ^8^ Interdepartmental Centre for Regenerative Medicine “Stefano Ferrari”, University of Modena and Reggio Emilia, Modena, Italy

**Keywords:** collagen, implant, E-beam, irradiation, rabbits, cornea

## Abstract

Sterilization of biodegradable, collagen-based implants is challenging as irradiation sterilization methods can alter their mechanical properties. Electron beam (EB) irradiation is a terminal sterilization method that has been used for biologically-derived implants. Here, recombinant human collagen type III-phosphorylcholine (RHCIII-MPC) hydrogels were irradiated with EB doses of 17, 19, or 21 kGy and their subsequent biocompatibility and ability to promote regeneration in rabbit corneas was evaluated. Unirradiated hydrogels stored in 1% chloroform in phosphate-buffered saline (C-PBS) were the controls. There were no significant differences between irradiated and non-irradiated samples in optical or physical properties (tensile strength, modulus, elasticity), or the ability to support cell growth. However, irradiated implants were more sensitive to high levels of collagenase than unirradiated controls and the C-PBS implants had increased cell growth compared to EB and controls at 72 h. Corneal implants e-beamed at 17 kGy or e-beamed and subsequently frozen (EB-F) to increase shelf-life showed no adverse biological effects of the irradiation. EB, EB-F, and C-PBS implanted corneas all rapidly re-epithelialized but showed mild neovascularization that resolved over 6 months. The regenerated neo-corneas were transparent at 6 months post-operation. *In vivo* confocal microscopy confirmed normal morphology for the epithelium, stroma, sub-basal nerves and unoperated endothelium. Histology showed that all the regenerated corneas were morphologically similar to the normal. Immunohistochemistry indicated the presence of a differentiated corneal epithelium and functional tear film. In conclusion, the e-beamed corneal implants performed as well as non-irradiated control implants, resulting in fully regenerated neo-corneas with new nerves and without blood vessels or inflammation that may impede vision or corneal function. Therefore, a complete validation study to establish EB irradiation as an effective means for corneal implant sterilization prior to clinical application is necessary as a next step.

## Introduction

Biomaterials are increasingly used as implants, but post-operative infections associated with the materials remain a significant complication. Implants are sterilized to minimize the risk of infection. However, those made from biodegradable, biologically-derived materials are often sensitive to conventional sterilization techniques and therefore, sterilization remains problematic (Zhang et al., 2006; Dai et al., 2016). We developed and successfully tested in clinical trials pro-regeneration biosynthetic corneas as prospective alternatives to human donor corneas for the treatment of corneal blindness. Our recombinant human collagen type III (RHCIII) implants successfully and stably stimulated regeneration of the corneal epithelium, stroma, and associated nerves after lamellar keratoplasty, without the need for sustained immunosuppression in a first-in-human study (Fagerholm et al., 2010, 2014). For use in patients with severe pathologies that put them at high risk of rejecting conventional donor transplantation, RHCIII implants incorporating a synthetic lipid polymer, 2-methacryloyloxyethyl phosphorylcholine (MPC) that suppresses inflammation, were successfully tested in high-risk patients with ulcerated and badly scarred corneas (Hackett et al., 2011; Islam et al., 2013, 2015; Kakinoki et al., 2014). In these first-in-human clinical studies, the implants were manufactured aseptically under Class 100 or ISO 5 conditions and stored in phosphate-buffered saline (0.1 M) containing 1% chloroform (C-PBS) to maintain sterility (Fagerholm et al., 2010, 2014; Islam et al., 2018). This storage solution required an extensive washing procedure to remove the chloroform before surgery, after which they were further washed in antibiotics before use to ensure their sterility.

For expanded clinical testing and future clinical application, an effective terminal sterilization procedure that allows the surgeon to open the vial to use the implants simply is needed. Ethylene oxide gas is used for sterilization but is toxic and carcinogenic (Mendes et al., 2007) and therefore not considered. Our corneal implants comprise mainly collagen. Like most complex proteins, collagen responds to heat (e.g., autoclaving) or irradiation by changing its physical or biological properties. With alterations in chemical and morphological structures, the associated biointeractive properties are also changed (Hoburg et al., 2010; Stoppel et al., 2014). Electron beam (e-beam) sterilization uses high-energy electrons that produce beams with a lower depth of penetration and high dose rate and is less stressful to materials than gamma irradiation, which has a low dose rate and high penetrability. E-beam at 15 and 25 kGy has been shown to preserve the mechanical properties of anterior cruciate ligament grafts (Hoburg et al., 2011). For collagen sponges, in particular, gamma irradiation at 2.5 Mrad has been shown to cause significant shrinkage (Noah et al., 2002). Nevertheless, low irradiation doses have been successfully used to sterilize biological materials such as collagen scaffolds (20 kGy), decellularized porcine super flexor tendon (15, 34 and 15 + 15 kGy), and decellularized porcine dermis (10, 25 and 40 kGy) (Dearth et al., 2016; Herbert et al., 2017; Monaco et al., 2017). The VisionGraft® is an acellular graft cornea gamma-irradiated at 17–23 kGy (Daoud et al., 2011; Chae et al., 2013; CorneaGen VisionGraft THE CLEAR CHOICE, 2021). Any radiation causing a decrease in the corneal melting temperature indicative of free-radical damage to the peptide backbone could affect the RHCIII fibrils present within RHCIII-MPC hydrogels. In contrast, e-beam has been successfully used to irradiate a number of different biomaterials: e.g., Kajii et al. e-beam irradiated at 15 and 40 kGy an octacalcium phosphate and collagen composite (OCP/Col) designed to promote bone regeneration as bioburden-spiked samples (Kajii et al., 2018). They found that while both doses sterilized the composites, the 15 kGy dose permitted more effective bone regeneration. In Proffen et al. (2015), extracellular matrix proteins including collagen were aseptically manufactured into scaffolds to improve anterior cruciate ligament repair. Subsequently, samples that were e-beam irradiated at 15 kGy maintained their sterility while non-irradiated scaffolds became contaminated with bacteria and fungi. These reports are in keeping with the industrial standard (ISO 11137-2), indicating that a 15 kGy irradiation dose can result in a log reduction of 10^6^ colony-forming units of bacteria and fungi when used on a material with a low initial bioburden (International Standards Organization, 2012).

The use of e-beam irradiation for the sterilization of medical devices requires process validation following ISO 11137-2:2012 (International Standards Organization, 2012). Prior to pursuing a very costly whole process validation, here, we evaluated the ability of low doses of e-beam irradiation to maintain the sterility of RHCIII-MPC corneal implants manufactured under low initial bioburden conditions, as an alternative to C-PBS. We also examined the effects of 17, 19, and 21 kGy of e-beam irradiation on the physical properties of the implants, and most importantly, biocompatibility and performance as corneal implants in rabbit models.

## Methods

### Implant Fabrication and Packaging

RHCIII-MPC implants were fabricated under aseptic conditions, as previously described (Islam et al., 2015). Briefly, 500 mg of 18% (w/w) aqueous solution of recombinant human collagen-III (Fibrogen Inc., San Francisco, CA) was buffered with 150 µL of 0.625 M 2-(N-morpholino)ethanesulfonic acid (MES; Sigma-Aldrich, Steinheim, Germany) buffer in a syringe mixing system. N-hydroxyl-succinimide (NHS; Sigma-Aldrich, Steinheim, Germany), 2-methacryloyloxyethyl phosphorylcholine (MPC; Paramount Fine Chemicals Co. Ltd, China), poly (ethylene glycol) diacrylate (PEGDA; Sigma-Aldrich, Steinheim, Germany), ammonium persulphate (APS; Sigma-Aldrich, Steinheim, Germany), N,N,N′,N′-tetramethylethylenediamine (TEMED; Sigma-Aldrich, Steinheim, Germany) and 1-ethyl-3-(3-dimethylaminopropyl) carbodiimide (EDC; Sigma-Aldrich, Steinheim, Germany) were sequentially added into the syringe mixing system followed by mixing at 0°C. The collagen primary amine:NHS:EDC molar ratio was 1:0.35:0.7 while the MPC:collagen ratio (w/w) was 1:2. PEG-DA:MPC ratio (w/w) equaled 1:3, APS:MPC ratio was 0.03:1 and APS:TEMED equaled 1:0.77. The implants were cast in 10 mm diameter, 500 μm thick, dome-shaped molds that are matched for corneal curvature and allowed to crosslink in a hydrated chamber at room temperature overnight. After demolding, the hydrogels were washed thoroughly in a phosphate bath and bottled in 0.1 M sterile phosphate-buffered saline (PBS). Implants were packaged in 10 ml of either 0.1 M PBS or in PBS containing 1% chloroform (C-PBS) in 10 ml sized vials. The vials were sealed with “tear-off’ aluminum crimp caps with 3 mm butyl/PTFE septa, sealed using a hand-crimper. After sealing, the vials were placed in double autoclave bags for irradiation.

For the *in vitro* e-beam dose-response study, implants were cast in dogbone-shaped molds, 0.5 mm thick, with a central test section with the dimensions 14 mm × 6 mm, and a grip area at each end of 6 mm × 10 mm.

For 17 kGy e-beam testing followed by third-party sterility testing, the implants were cast as 12 mm wide, 350 and 500 μm thick corneal-shaped implants with 3 mm concave curvature. Similar implants were cast for rabbit *in vivo* testing at 350 μm.

### E-Beam Irradiation and Ability to Retain Sterility

To determine an optimal e-beam dose, three implants per group were sent for e-beam sterilization at 17, 19, and 21 kGy (Sterigenics, Espergarde, Denmark). A dosimeter packet was placed with each vial during irradiation to measure the absorbed dose. The applied radiation dose was very precisely controlled with an acceptable dose deviation of ±0.1 kGy. Another three control implants were stored in C-PBS.

The initial bioburden and endotoxin levels of the implants were assessed by the sterilization provider following DS/EN ISO 11737-2 (Sterigenics, Espergaerde, Denmark) prior to e-beam. Sterility and endotoxin levels were assessed for the 17 kGy dose following DS/EN ISO 11737-2 by a second independent third party (APL, Stockholm, Sweden). The sterility test was conducted following Ph. Eur. 2.6.1 Sterility, using the direct inoculation method (Shetty et al., 2018). Human cornea-shaped and sized implants (10 mm diameter, 500 µm thick curved hydrogels) were irradiated at 17 kGy. Irradiated implant samples were then immersed directly into tryptone broth and incubated at 28–32°C for 14 days. During this time, the contents of the containers were examined for evidence of microbial growth. If turbidity were observed, confirmation of growth or no growth is done by sub-culturing on tryptone soya agar (TSA) plates at 30–35°C for an additional 7 days. The amount of bacterial endotoxin in the irradiated hydrogels was tested following Ph. Eur. 2.6.14 Bacterial endotoxins, using the gel clot method that detects and quantifies the amount of toxin present by the clotting of an amoebocyte lysate from the horseshoe crab (European Directorate for the Quality of Medicines & HealthCare, 2014).

#### Sterility of Controlled Bioburden Corneal Samples

**FIGURE 1:**
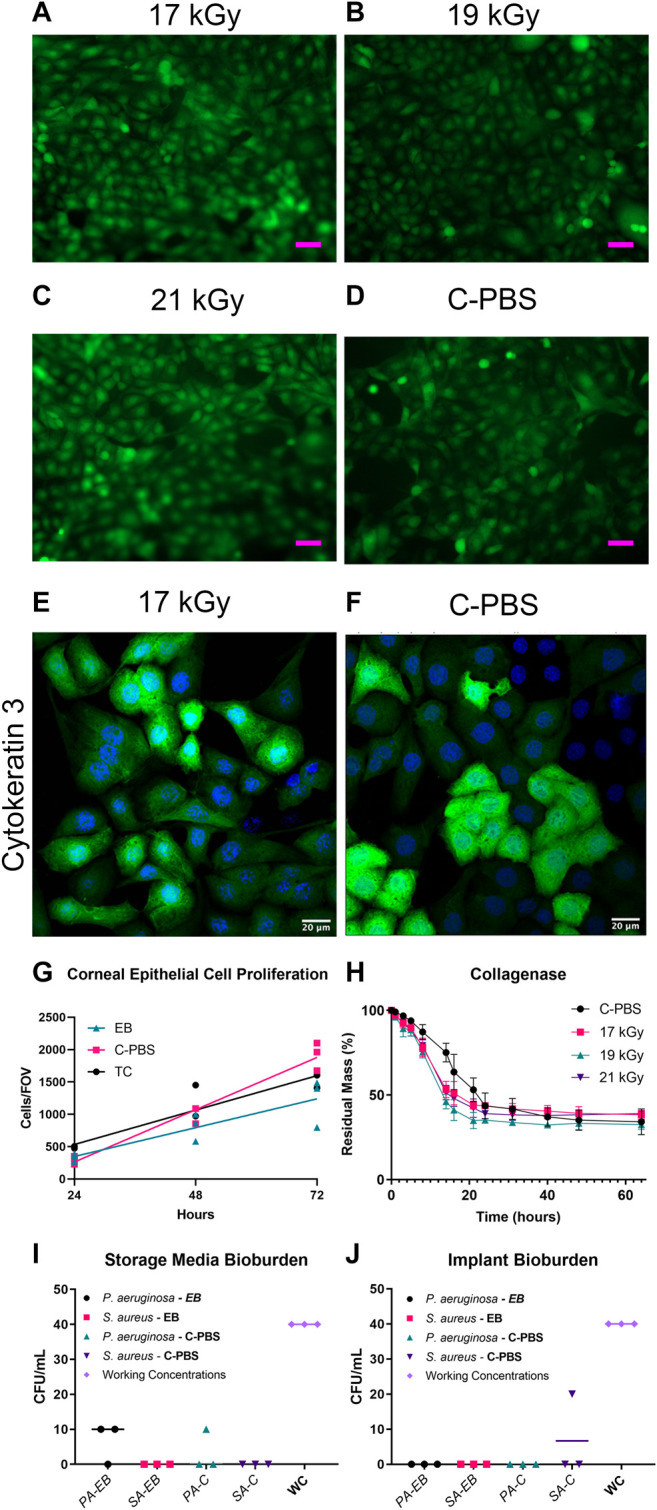
GFP-HCEC cells on e-beam irradiated and unirradiated RHCIII-MPC implants showed confluence by day 4 in all cultures. **(A)** 17 kGy, **(B)** 19 kGy, **(C)** 21 kGy, **(D)** Unirradiated (C-PBS) (*n* = 4, all groups). HCECs cultures on 17 kGy e-beamed samples **(E)** compared to C-PBS treated hydrogels **(F)** showed similar cytokeratin 3 staining. **(G)** Quantification of corneal epithelial cell proliferation on implants that have been e-beamed, stored in C-PBS, or on tissue culture (TC) plastic. Lines show linear regression of the data. **(H)** Collagenase degradation of the e-beamed materials demonstrating that e-beam changed the rate but not the extent of collagenase degradation of irradiated E-beamed RHCIII-MPC (*n* = 4, all groups). Post-e-beam bioburden measured in the storage media in the *P. aeruginosa* and *S. aureus*-spiked implants **(I)** and direct culture of the corneal implants **(J)** showed a significant reduction from the 400 CFU/ml (*n* = 4, all groups).

The samples sent for the initial bioburden analysis described above did not contain any microorganisms, so they could not be used to establish the dose based on the procedure described in DS/EN ISO 11737-2, which requires the medical device to have a positive bioburden during the initial tests. Therefore, to determine efficacy of e-beam irradiation in decreasing bioburden, corneal samples were manufactured and intentionally inoculated with known bioburden. The efficiency of sterilization methods was evaluated against Gram (+) and Gram (-) bacteria, *Staphylococcus aureus* and *Pseudomonas aeruginosa,* respectively. The individual implant was placed in a 10 ml PBS containing vial. *Staphylococcus aureus* (∼400 CFU) was added to half of the vials (*n* = 6) and the rest of the vials were treated with *Pseudomonas aeruginosa* (∼400 CFU) (Figures 1A,F)*.* From each bacteria group, half of the vials (*n* = 3) were sent for E-beam irradiation (17 kGy, Nutek Bravo, Hayward, CA 94545, United States) and to the rest of the vials (*n* = 3) 1% chloroform was added. After irradiation, the vials were returned to the lab and tested for bacterial viability. For double confirmation of the sterility, two sets of studies were performed with the irradiated and chloroform treated vials. In one study set, 100 μL of the vial storage solution were streaked over tryptic soy agar plates and monitored for bacterial growth. In the other study set, the implants from the vials were transferred to another sterilized vial containing 2 ml tryptic soy broth (TSB) media (Teknova Inc., Hollister, CA 95023, United States) media. These vials were incubated overnight with shaking (80 rpm). Then the TSB media from the vials were streaked over tryptic soy agar plates and monitored for bacterial growth. After 24 h, the total CFU for both bacteria were counted.

### Materials Testing

#### Mechanical and Thermal Properties

Three dogbone-shaped hydrogels receiving 17, 19, or 21 kGy of irradiation or C-PBS stored controls were examined. Tensile strength, Young’s modulus, and elongation at break were measured using an Instron Universal test machine (Biopuls 3343, High Wycombe, United Kingdom). These measurements were carried out underwater immersion at 37°C. The crosshead speed was 10 mm•min^−1^ and the load cell was 50 N. All the samples broke at the waist of the dogbone-shaped sample.

The thermal properties of the hydrogels were measured using differential scanning calorimetry (DSC). The denaturing temperature was determined using a Cellbase DSC (Instrument Specialists Inc, Twin Lakes, United States), measured in the heating range of 8–80°C at a scan rate of 8°C min^−1^. Approximately 5–10 mg of the hydrogels were weighed after removing the surface water and hermetically sealed in an aluminum pan to prevent material dehydration. T_max_ of the curve of heat flow (W/g) versus temperature (°C) gives the denaturing temperature.

#### Optical Properties

Light transmission and backscattering measurements of e-beam irradiated and C-PBS treated implants (*n* = 3 per group) were carried out at room temperature using a custom-built instrument, as previously reported (Liu et al., 2008).

#### Biodegradation Study

**FIGURE 2:**
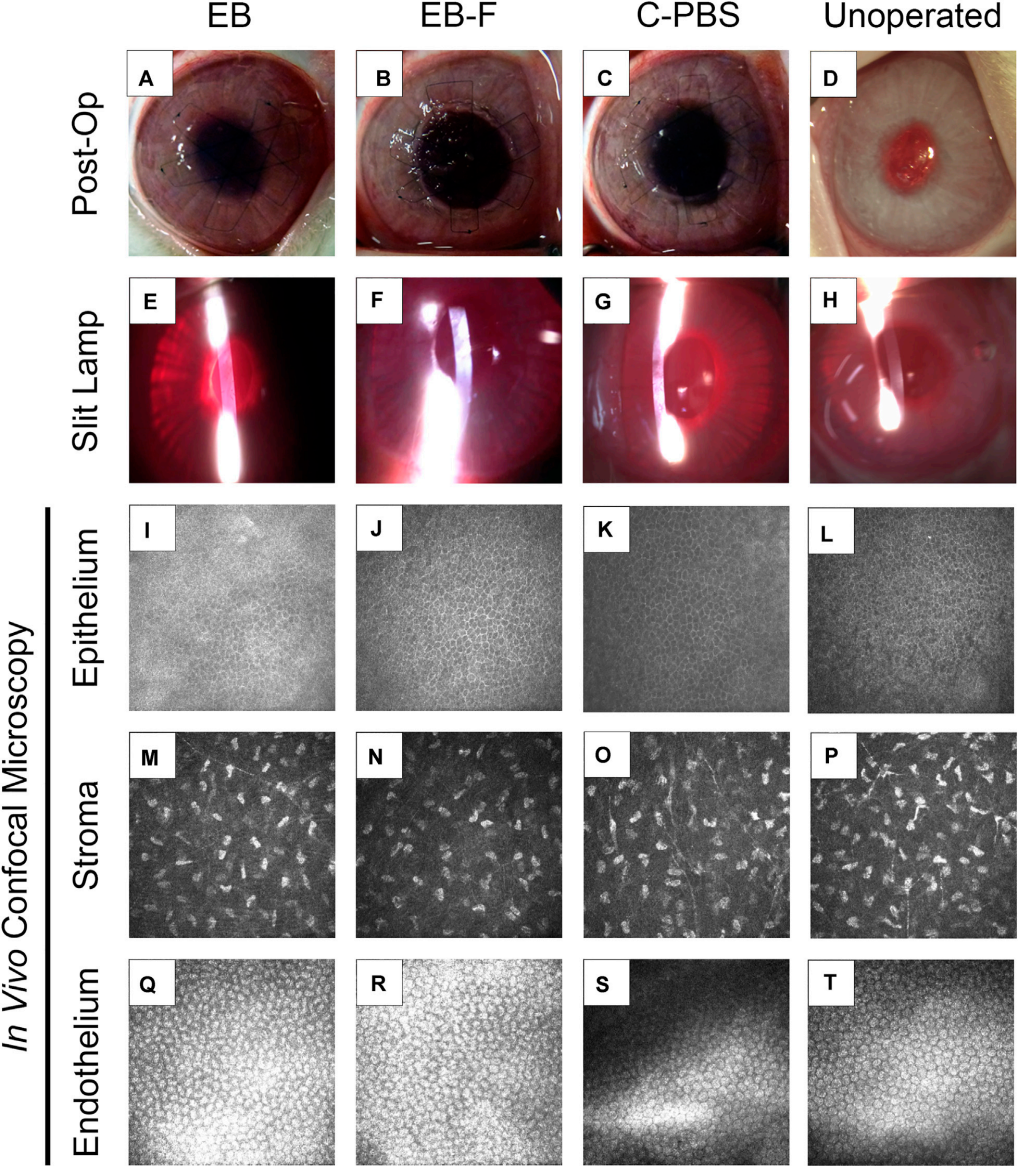
RHCIII-MPC implants that had been sterilized with e-beam at 17 kGy irradiation in phosphate-buffered saline (*n* = 4) **(A,E,I)**, irradiated and then stored frozen at −80°C after withdrawal of saline (*n* = 4) **(B,F,J)** or in phosphate buffered saline (PBS) containing 1% chloroform (*n* = 4) **(C,G,K)** after grafting into rabbit corneas in comparison to unoperated eyes (*n* = 12) **(D,H,L)**. Slit lamp images at 6 months post-operation **(E–H)** and corresponding *in vivo* confocal microscopy (IVCM) images at these times **(I–T)**. The IVCM images show the regenerated epithelium **(I–L)** and stroma **(M–P)**, as well as healthy endothelium beneath the implant area **(Q–T)**. Unoperated (*n* = 12), unirradiated (*n* = 4), and irradiated (*n* = 4).

Collagenase from *Clostridium histolyticum* (Sigma-Aldrich, MO, United States) was used to evaluate the biodegradation of irradiated and unirradiated hydrogels. Approximately 15 mg of each hydrogel (*n* = 3 per group) were cut out and placed into 0.1 M Tris-HCl buffer [tris(hydroxymethyl)aminomethane hydrochloride (Merck KGaA Darmstadt, Germany) containing 5 mM calcium chloride and 5 U/ml collagenase. The collagenase solution was refreshed every 8 hours. At different time points (Figure 2A), each sample was weighed after blotting off surface water. The percentage of residual weight was calculated using the following equation: Residual mass% = W_t_/W_o_ %, where W_t_ is the weight of hydrogel at a particular time point and W_o_ is the initial weight of the hydrogel.

### *In vitro* Biocompatibility

To evaluate the effect of e-beam irradiation on cell growth, green fluorescence protein (GFP) transfected immortalized human corneal epithelial cells (GFP-HCECs) were seeded onto hydrogels that were e-beam irradiated at doses of 17, 19, and 21 kGy (Islam et al., 2015). Controls consisted of C-PBS incubated hydrogels. All the hydrogels were trephined into 6 mm discs to fit into the wells of a 96-well plate. Five thousand GFP-HCECs were seeded onto each hydrogel sample and maintained in Keratinocyte Serum-Free Medium (KSFM; Life Technologies, Invitrogen, Paisley, United Kingdom) containing 50 μg/ml bovine pituitary extract and 5 ng/ml epidermal growth factor in a 37°C incubator. The medium was changed on every alternative day. Images of cultured cells were taken at different time points using a fluorescence microscope (AxioVert A1, Carl Zeiss, Göttingen, Germany).

To ensure that the irradiated hydrogels supported cell differentiation, HCECs were cultured on 5 mm discs of 17 kGy and C-PBS treated hydrogels in a 96 well plate with a seeding density of 1000 cells/well. The cells were fixed with 4% paraformaldehyde and stained with cytokeratin 3 (1:100, ab77869, AbCam, United Kingdom) with goat anti-Rabbit, Alexa Fluor® 488 secondary (1:1000, A11034, Invitrogen, United States). The cells were maintained in KSFM in a 37°C incubator for 5 days. The hydrogels were removed from the wells and mounted on slides with coverslips for visualization using a fluorescent microscope (Zeiss AxioImager Z2, Carl Zeiss, Göttingen, German).

### *In vivo* Evaluation in Rabbit Corneas

This study was conducted in compliance with the Swedish Animal Welfare Ordinance and the Animal Welfare Act, and with ethical permission from the local ethical committee (Linköpings Djurförsöksetiska Nämnd). Three groups of curved RHCIII-MPC implants 6.25 mm in diameter and 350 µm thick were tested. These were either been e-beam irradiated at 17 kGy, irradiated, and then frozen at −80°C after PBS removal, or maintained sterile in C-PBS. Four rabbits were used per group as the primary outcome measures are semi-quantitative and the sample sizes needed to perform equivalence studies exceed the capacity of standard animal facilities. One implant from each group was grafted into the right cornea of a New Zealand rabbit (weight 3.5–4 kg) by anterior lamellar keratoplasty (ALK), *n* = 4 per group. Rabbits were anesthetized with xylazine (Rompun; Bayer, Gothenburg, Sweden) and ketamine (Ketalar; Parke-Davis, Taby, Sweden). Each rabbit cornea was cut centrally with a 6 mm diameter Baron Hessberg trephine set to a depth of 300 µm. The corneal tissue was then dissected lamellarly with a diamond knife and removed. A 6.25 mm diameter implant was placed into the wound bed and anchored with three 10/0 nylon overlying sutures. Animals were given antibiotics in the form of a 1% fucithalmic ointment (Fucithalmic; Leo Pharma AB, Malmö, Sweden) topically 2 times daily during the first week after the surgery. No immunosuppression was used. Sutures were removed at 1-month post-operation.

Clinical examinations were performed daily on each animal for up to 7 days post-operative, and then at 1, 3, and 6-months post-operation. Slit-lamp biomicroscopy was used to evaluate the implants for optical clarity/haze and any inflammation (as indicated by excessive conjunctival redness, swelling compared to the unoperated contralateral control eye) or neovascularization using a modified MacDonald-Shadduck scoring system (Altmann et al., 2010). Other tests included intraocular pressure (IOP) measurements, Schirmer’s strip test for tear production, fluorescein staining to access epithelial integrity, ultrasound pachymetry (Tomey SP 3000, Tomey, Inc., Japan) to check the corneal thickness and aesthesiometry to assess corneal touch sensitivity (Cochet-Bonnet aesthesiometer, Luneau Oftalmologie, France).

Pre-operatively and at the 6-months follow-up, both corneas of each rabbit were examined by *in vivo* confocal microscopy (IVCM) (ConfoScan3, Nidek, Japan) to image epithelial coverage in-growth of stromal cells, nerves, and any blood vessels or immune infiltrate into the implants. A total of 2106 IVCM images were analyzed from 16 eyes of eight rabbits. Nerve count analysis was performed according to Lagali et al. (Lagali et al., 2007). All images with nerves or nerve fiber bundles (referred to collectively as nerves) were identified. For identification purposes, nerves were defined as bright, slender, straight, or branching structures, as substantially uniform in intensity along their length and width, and as having a marked contrast difference from the background intensity level. The following parameters were noted for each image: corneal depth location and the number of nerves present. A total of 302 images with nerves were analyzed from all the groups. To describe the location of corneal nerves, four corneal zones were defined: 20–50 μm below the epithelial surface, representing the nerves of the subbasal nerve plexus at the basal epithelial and subepithelial regions; sixty to 100 μm below the epithelial surface, representing the most anterior stromal region; 110–150 μm below the epithelial surface, representing the deep anterior stroma; 160 μm and deeper—mid and deep corneal stroma. The outcome measures used in this study consisted of the total number of nerve branches compiled within each depth zone and the total number of nerves per cornea.

### Histopathology and Immunohistochemistry

Rabbit corneas were excised with a 3–4 mm rim of sclera around them, rinsed in 0.1 M phosphate-buffered saline (PBS), and then fixed in 4% paraformaldehyde in PBS. They were either processed for paraffin embedding or frozen in optimal cutting temperature (OCT) compound.

Paraffin embedded sections were used for routine hematoxylin-eosin staining for histopathological examination. Paraffin sections were also used for histochemical staining with picrosirius red and alcian blue (at pH 2.5) to visualize collagen and glycosaminoglycans within the corneal extracellular matrix. FITC-conjugated Ulex europaeus agglutinin (UAE) was used for mucin detection. All samples were deparaffinized in xylene followed by alcohol washes from 100 to 70% ethanol to re-hydrate the sections. For the collagen stain samples were incubated in Alcian blue (A5268-10G, Sigma-Aldrich) for 20 min, washed in running tap water for 10 min, then incubated in picrosirius red solution (ab150681, AbCam, United Kingdom) for 45 min. The samples were washed in acidified water for 5 min prior to dehydration and mounting in Permount (SP15-100, FisherSci). These samples were imaged on a Zeiss AxioImager Z2 with an AxioCam MRc color CCD camera (Carl Zeiss, Oberkochen, Germany). For mucin, the sections were blocked in 0.1 M Tris-buffered saline containing 5% normal goat serum and 0.01 mg/ml saponin. The slides were incubated in UAE at a concentration of 1:100 overnight at 4°C. They were washed and stained with DAPI prior to mounting in Vectashield Vibrance mounting medium (Vector Laboratories, Inc., Burlingame, CA). Mucin was imaged using a Zeiss LSM 880 with a ×20 water immersion objective (Carl Zeiss, Oberkochen, Germany).

Frozen sections were prepared for immunohistochemical staining with antibodies against cytokeratin 3 and cytokeratin 12 (2Q1040, ab68260, Abcam, United Kingdom) at a 1/50 dilution. Seven-micron frozen sections irradiated, irradiated, and frozen and non-irradiated, implanted corneas, as well as their corresponding unoperated contralateral controls, were used and mounted on glass slides. Samples were fixed with cold acetone (10 min, −20°C), air dried, immersed in PBS, and then blocked with 5% goat serum in PBS with 0.1% Tween 20 (blocking solution) for 60 min at room temperature. Successively, incubation with all the primary antibodies diluted with the blocking solution was carried out overnight at 4°C. All slides were washed in PBS with 1% Tween 20 (PBS-T) and then incubated with goat anti-mouse Alexa 488 (Jackson Immuno Research Laboratories, Inc., West Grove, PA) diluted 1:1000 with the blocking solution for 60 min at room temperature. After washing in PBS-T, the slides were dehydrated and mounted with Vectashield mounting medium with DAPI (Vector Laboratories, Inc., Burlingame, CA). An LSM-700 Zeiss upright confocal microscope (LSM700, Carl Zeiss, Oberkochen, Germany) with a ×20 objective was used for capturing images.

### Statistical Analyses

Statistical analyses were conducted using GraphPad Prism 8 (GraphPad Software Inc., La Jolla, CA, United States). Data are presented as mean ± SD unless otherwise indicated. For all tests within this study, a *p* ≤ 0.05 was considered statistically significant. Data were checked for normality using a Shapiro-Wilkes test where appropriate (*n* < 50). One- and two-way ANOVA with Tukey/Tamhane’s T2 post-hoc analyses was used to check between-group differences for data with a normal distribution, including optical, mechanical, and thermal data. Collagenase degradation was analyzed using non-linear regression to determine if the degradation rate differed between the irradiated and unirradiated samples. A one-phase exponential decay model was compared to a sigmoidal curve to assess the best fit based on graphical analysis of the raw data. The comparison resulted in a sigmoidal curve being preferred for all data sets. The data were fitted with a sigmoidal curve and tested to determine if one curve fit all data and if the best-fit values of selected unshared data points differed between data sets. Clinical data were analyzed using a Kruskal-Wallis test with Dunn’s multiple comparison test for post-hoc tests.

Corneal IVCM images were sorted according to the eye, depth zone, and whether exposed to e-beam or not. Paired sample t-tests were used to determine significant differences between nerve numbers, corneal thickness, and corneal aesthesiometry in control versus surgical corneas in each depth zone. Student t-tests were used for comparisons between irradiated and non-irradiated samples.

## Results

### E-Beam Irradiation and Sterility

The irradiation of the dog bone shaped hydrogels at 17 ± 0.1 kGy, 19 ± 0.1 kGy and 21 ± 0.1 kGy, and cornea-shaped samples at 17 ± 0.1 kGy was confirmed. Independent analyses showed that after the 14-days sample immersion in broth, the 17 kGy-irradiated implants showed no microbial growth, indicating that the samples maintained their sterility (Sterigenics and APL, Stockholm, Sweden). The endotoxin test results showed that the implants were compliant with the <0.5 EU/mL cut-off requirement for implantable medical devices (U.S. Department of Health and Human Services Food and Drug Administration, 2012).

For the controlled bioburden samples, e-beam was shown to be effective against both Gram + and – bacteria (Figures 1H,I). Zero CFU was observed from storage media of *Staphylococcus aureus* added vials, irrespective of sterilization methods (Figures 1H,I). Two *Pseudomonas aeruginosa* treated vials showed 1 CFU each when irradiated (Supplementary Figure S1), whereas only one vial from the chloroform sterilized group of *Pseudomonas aeruginosa* showed 1 CFU per plate (Supplementary Figure S1). Implants soaked in TSB confirmed the sterility of the implants (Supplementary Figure S1). One chloroform treated *Staphylococcus aureus* vial showed 2 CFU per plate (Supplementary Figure S1). Vials that were not explicitly mentioned carried zero observed CFU.

### Materials Properties

#### Optical and Mechanical Properties

A summary of the mechanical, optical and thermal stability testing results is given in Table 1. There were no significant group differences for any mechanical or optical properties between the C-PBS and e-beam doses. One-way ANOVA of the thermal stability measurements obtained using DSC showed an overall significant difference (*p* = 0.02). There were significant differences between 17 and 21 kGy (*p* = 0.02), as well as 19 and 21 kGy (*p* = 0.03); however, no between group differences were observed between the unirradiated and irradiated groups (*p* = 0.75).

**TABLE 1:** Comparison of physical properties of e-beam irradiated and unirradiated corneal implants. Mechanical and optical properties of irradiated and unirradiated implants.

	C-PBS (n = 3)	17 kGy (n = 3)	19 kGy (n = 3)	21 kGy (n = 3)	*p*-Value
Optical Properties					
Transmission (%)	88 ± 1.9	84 ± 3.8	88 ± 4.6	88 ± 7.8	0.7
Backscatter (%)	1.6 ± 0.4	1.2 ± 0.7	0.03 ± 0.06	1.2 ± 1.4	0.2
Mechanical Properties					
Tensile Strength (MPa)	0.3 ± 0.1	0.3 ± 0.04	0.2 ± 0.1	0.3 ± 0.3	0.7
Elongation at break (%)	12.15 ± 0.84	12.17 ± 0.40	10.15 ± 2.33	11.48 ± 4.39	0.8
Young’s modulus (MPa)	3.6 ± 0.8	3.7 ± 0.6	2.9 ± 0.5	4.1 ± 3.2	0.9
Thermal Stability					
Denaturation Temperature (^o^C)	53 ± 1.7	51 ± 2.3	51 ± 2.0	56 ± 0.5	0.02

### Response to Collagenase Enzyme

The collagenase biodegradation study was conducted to compare the stability of the hydrogels in response to enzymatic degradation (Figure 1G; Table 2). Each data set was fitted with a sigmoidal curve with a top value constrained at 100% to account for total solid content mass at the beginning of the assay. A test for one curve for all data sets was rejected (*p* < 0.0001)), indicating that each curve was different. The hill slope of the irradiated implants was steeper than the C-PBS implants, demonstrating an initial increased rate of degradation in the presence of collagenase within the first 24 h, before leveling out.

**TABLE 2:** Comparison of physical properties of e-beam irradiated and unirradiated corneal implants. Sigmoidal regression of irradiated and unirradiated implants after collagenase treatment.

	C-PBS (*n* = 3)	17 kGy (*n* = 3)	19 kGy (*n* = 3)	21 kGy (*n* = 3)	*p*-Value
Top	= 100	= 100	= 100	= 100	N/A
Bottom	36 ± 1.6	41 ± 0.9	33 ± 0.8	39 ± 0.7	<0.0001
IC50	15 ± 0.6	10 ± 0.4	9.6 ± 0.3	10 ± 0.3	<0.0001
Hill Slope	-0.09 ± 0.01	-0.13 ± 0.01	-0.14 ± 0.01	-0.13 ± 0.01	0.002
Span	= 64	= 59	= 67	= 61	N/A
R square	0.96	0.98	0.99	0.99	N/A

Data is reported as mean ± SE. Top value was constrained to 100.

### *In vitro* Cell Biocompatibility

Both unirradiated and irradiated RHCIII-MPC hydrogels at all three doses supported the attachment and proliferation of GFP-HCEC cultured on them (Figures 1A–F). Cultures of GFP-HCEC reached confluence at day four on all hydrogels. The cells had a higher rate of proliferation on C-PBS treated materials, than EB or tissue culture plastic (TC) over 72 h (Supplementary Table S1). Cytokeratin three staining of primary HCECs cultured on the 17 kGy and C-PBS materials showed that both hydrogels support terminally differentiated corneal epithelial cells (Figures 1E,F).

### Effect of E-Beam Sterilization on Implant Performance in Rabbit Corneas

#### Follow-Up Over 6 months

Post-surgical slit lamp examination of the implanted corneas showed no excessive redness or swelling in irradiated implants compared to non-irradiated control samples. All implants were stably incorporated over the surgical period without the use of immune suppressive eye drops.

**FIGURE 3:**
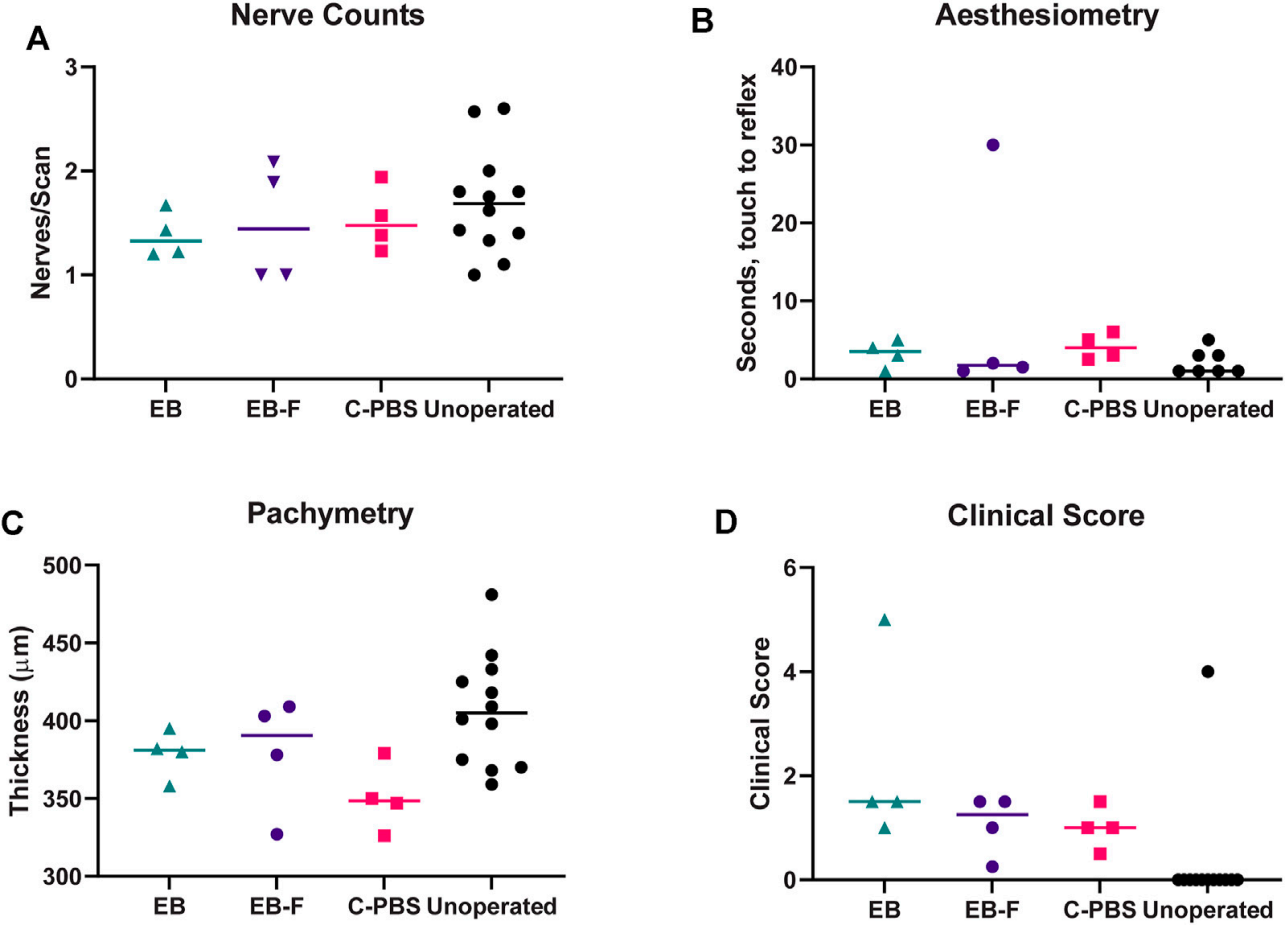
Innervation, thickness and microscopy of the regenerated cornea. **(A)** Number of nerve fibers per central IVCM corneal scan in RHCIII-MPC implanted corneas at 6 months post-implantation. **(B)** Results of Cochet-Bonnet corneal aesthesiometry at 6 months after surgery. **(C)** Corneal thickness at 6 months post-implantation. **(D)** Composite McDonald-Shadduck clinical score at 6 months post-implantation. Unoperated (*n* = 12), unirradiated (*n* = 4), and irradiated (*n* = 4).

Full epithelial coverage of the implants was completed within the first week post-surgery, as demonstrated by the exclusion of sodium fluorescein, when the dye was applied. The healing process was accompanied by mild neovascularization in all implanted animals. However, the neo-vessels gradually resolved. At 6 months post-implantation, no or very few ghost vessels remained. Mild subepithelial haze (grade 0.5–1) was observed in all rabbits throughout the follow-up period regardless of the sterilization method (Table 3), but all implanted grafts remained transparent (Figures 2A–C). One rabbit in the irradiated group experienced significant subepithelial fibrosis and haze in both the operated and unoperated eyes, leading to a significant outlier in the statistical analysis (Figure 3). Measurement of corneal thickness in the central zone at 6 months after surgery by pachymetry revealed that the corneas implanted with unirradiated implants were thinner than unoperated corneas. Still, the irradiated implants were not significantly different from either group (Table 4).

**TABLE 3:** Slit lamp evaluation performed at 6 months post-operative.

Outcome	Unoperated (median) (*n* = 8)	C-PBS [median, (mean rank diff. To UO, significance)] (*n* = 4)	17 kGy [median, (mean rank diff. To UO, significance)] (*n* = 4)	K-W
Corneal Opacity Severity	0	1 (-5.6, ns)	1 (-6.9, *)	8.4, *p* = 0.003
Corneal Opacity (Area)	0	1 (-5.6, ns)	1 (-6.9, *)	8.4, *p* = 0.003
Corneal Vascularization	0	0 (-2.0, ns)	0 (-2.0, ns)	3.0, *p* = 0.5
Conjunctival Congestion	0	0	0	N/A
Conjunctiva Chemosis and Swelling	0	0	0	N/A
Corneal Staining	0	0	0	N/A

The exam was performed by two independent raters with an inter-rater reliability score of *κ* = 0.594, so the median score, rounded up, was used for all comparative analysis. Clinical score is reported as the median group score. Groups were compared using a Kruskal-Wallis test, with a Dunn’s multiple comparison correction for between group analyses.

**TABLE 4:** Corneal thickness measurements of the implantation area of operated and non-operated eyes at 6 months post-operation by pachymetry.

Group	Corneal Thickness M ± SD	Mean rank Difference to unoperated
17 kGy (*n* = 4)	378 ± 18.8	2.5
C-PBS (*n* = 4)	352 ± 21.8	7.5*
Unoperated (*n* = 8)	397 ± 27	-

Statistical significance (*p* ≤ 0.05) of operated eyes from healthy, unoperated control eyes was determined using a Kruskal-Wallis test (6.6, *p* = 0.03) with a Dunn’s multiple comparison between experimental and unoperated corneas.

### *In vivo* Confocal Microscopy

*In vivo* confocal microscopy performed at 6 months post-surgery showed that the epithelial and stromal layers had regenerated as in all previous RHCIII-MPC grafts in various species. The morphology of epithelial and stromal cells in irradiated, C-PBS, and control untreated corneas were similar. Both e-beamed and C-PBS hydrogel implanted corneas were re-innervated (Figures 2I–L, Figures 3A). Nerve counts made from IVCM images revealed that sterilization with e-beam and or 1% chloroform solution did not influence the rate of nerve regeneration (Figure 3A). Cochet-Bonnet aesthesiometry showed no differences between nerve sensitivity in the corneas (Figure 3B).

### Histopathology and Immunohistochemistry

**FIGURE 4:**
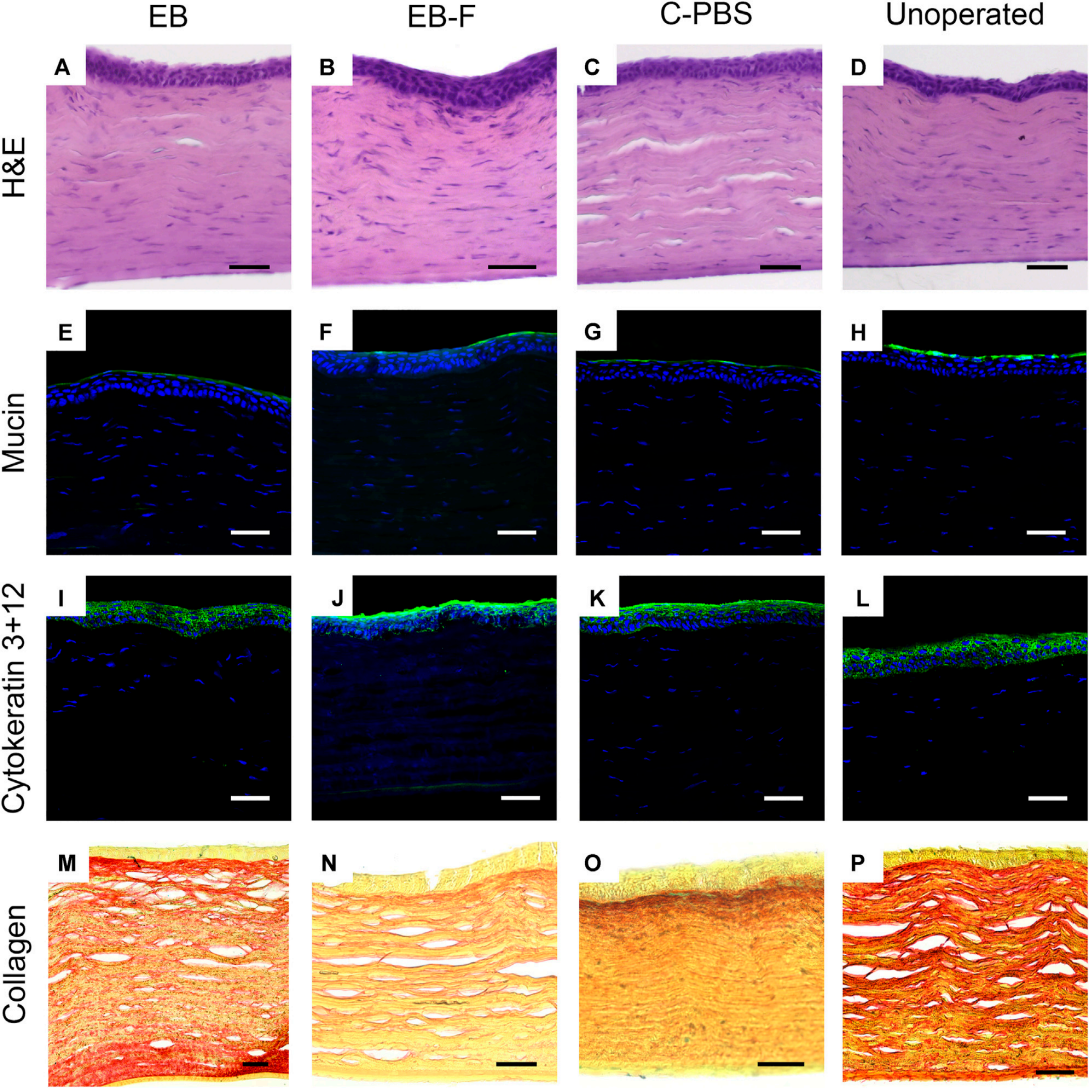
Microscopy of representative sections from untreated corneas (*n* = 12), e-beam irradiated corneas (*n* = 4), e-beamed and frozen corneas (*n* = 4) and C-PBS stored corneas (*n* = 4). **(A–D)** Hematoxylin-eosin stained sections showing morphology of corneas with regenerated epithelia and stromas; endothelia and posterior stroma was untreated. **(E–H)** Ulex europaeus agglutinin staining showing a green fluorescently labelled tear film in all samples. **(I–L)** Cytokeratin 3 + 12 (green) staining of regenerated epithelia. **(M–P)** Picrosirius red-alcian blue stained samples showing red stained collagen fibrils arranged in lamellae in the stroma of all samples. White and black scale bars 50 μm, yellow scale bars 25 μm.

Histopathological examination of H&E sections of the neo-corneas that regenerated after implantation with e-beam irradiated, e-beam irradiated and frozen, and C-PBS samples all had stratified epithelia and lamellate stroma with flattened cells, similar to that of the untreated, healthy contralateral corneas (Figures 4A–D). No significant differences in epithelial thickness were noted. The sections were also free from any infiltrating immune cells.

Immunohistochemistry showed that like healthy control corneas, the regenerated neocorneas from both irradiated and unirradiated corneal samples stained positively for a regenerated mucin layer (Figures 4E–H). The epithelia of all samples were stained with cytokeratins 3 and 12, indicating a mature, fully differentiated structure (Figures 4I–L). Picrosirius red staining of collagen fibrils in the regenerated neo-corneal stromas of e-beamed and frozen corneas retained a regular lamellar organization like C-PBS and the untreated healthy corneas. There was no significant alcian blue staining in any of the samples (Figures 4M–P).

## Discussion

E-beam is a widely accepted method for sterilization and has been effectively used for terminal sterilization to eliminate any microbial, fungal, or viral contamination that may have been introduced during the manufacturing process. E-beam sterilization is governed by the ISO standards 11137 and 13409 and uses very high-energy electrons that directly destroy bioburden. The high-energy electrons also collide with other local electrons, generating secondary electrons with sufficient energy to destroy bioburden.

While RHCIII-MPC implants have been manufactured aseptically and stored with 1% chloroform to maintain sterility in clinical trials with small cohorts of patients, for routine clinical use, a more repeatable and controlled process that gives a high assurance of sterility is needed. E-beam irradiation at 17, 19, and 21 kGy did not significantly alter the optical or mechanical properties of the RHCIII-MPC implants when compared to unirradiated controls. The lack of observed changes may be attributed to the EDC crosslinking, as it has been reported that EDC crosslinked materials are subject to radioprotective effects during e-beam irradiation (Seto et al., 2008).

The irradiated samples, however, showed an initial difference in the rate of collagenase degradation, but after 24 h, the rates between the 17 kGy irradiated and C-PBS samples were similar. This suggested that the implants could have an altered rate of initial remodeling within the body after implantation, in keeping with the observation that e-beam increases percent weight loss in ECM-based substrates (Grimes et al., 2005). However, as seen in the follow-on *in vivo* studies as discussed below, there were no obvious biological effects over the 6-month implantation period in the rabbit corneas.

The dose range study established that the minimum e-beam dose tested, 17 kGy, was effective at maintaining the sterility of the aseptically fabricated implants. As the goal of these implants is to act as a substrate for the complete remodeling of the cornea during the regenerative process, an irradiation dose that may increase the rate of degradation of the RHCIII-MPC matrix may be unsuitable to promote the formation of a cornea of appropriate thickness and mechanical strength; therefore, the higher 19 and 21 kGy doses were excluded from further study.

After 17 kGy irradiation, there was no bacterial growth on the irradiated hydrogel samples after 14 days of immersion into bacterial growth medium, confirming the ability of the samples to retain sterility. The spiking of the samples with a common Gram positive and Gram negative bacterium, *P. aeruginosa* and *S. aureus*, respsctively, showed that e-beam irradiation was able to significantly reduce the CFU and there was no CFU in the actual irradiated hydrogel samples. The very low CFU in the saline could have been introduced during the post-irradiation testing from the environment. For a formal follow-up validation study as a next step, a full dose mapping and VDmax15 procedure would be required.

There were no significant differences in the long-term performance between irradiated and unirradiated implants for any of the outcome measures studied. Freezing of irradiated e-beam samples did not significantly alter their performance *in vivo.* The differences in thickness observed between all the groups of implanted, regenerated neo-corneas and their contralateral unoperated eyes were non-significant. The thickness differences were most likely due to growth of the unoperated cornea as rabbits matured, compared to the catching-up required in the operated eyes. Both classes of implants resulted in successful re-epithelialization, demonstrating that the irradiated RHCIII-MPC matrix retained the critical biochemical or structural properties required to support the attachment and migration of limbal epithelial stem cells from the periphery of the cornea over the implant, and their subsequent stratification to re-establish a multilayered epithelium. The presence of differentiation markers, cytokeratins 3 and 12, plus mucin in the corneal explants, without changes in intensity and thickness, indicates that the regenerated epithelium in corneas grafted with both irradiated and non-irradiated implants was fully differentiated and could secrete mucin, i.e., was fully functional. The picrosirius red staining of collagen fibrils showed a lamellar arrangement in all the regenerated stromas. Hence, the observed changes in collagenase degradation profiles between the C-PBS and 17 kGy implants did not have a biological effect on the ability of the implants to stimulate regeneration of a morphologically accurate and functional epithelium.

Equivalent nerve counts confirmed functional innervation of the regenerated neo-corneas and blink response in both grafts compared to unoperated controls. We also found that freezing of e-beamed samples at −80°C did not result in a loss in the ability of RHCIII-MPC hydrogels to promote regeneration of corneal epithelium, stroma, and nerves.

E-beam irradiation has been used in the sterilization of commercially available ECM-based biomaterials in clinical applications; including the artificial skin, Integra®, which is made from collagen and glycosaminoglycans (Mattern et al., 2001). A dose of 20 kGy was used, but the matrices were irradiated dry. A lower dose such as ISO 11137-2:2015 Method VD_max_15, however, is a validated dose that has been used for e-beam sterilization (International Standards Organization, 2012). In this study, a slightly higher dose of 17 kGy irradiation of RHCIII-MPC in PBS maintained sterility of the implants whole preserving their ability to promote regeneration. In the future, the verification of the safety and efficacy of this dose will allow for sterility validation following ISO 11137-2:2015 Method VD_max_15.

In conclusion, we have shown that an e-beam dose of 17 kGy can be used to maintain the sterility of aseptically fabricated RHCIII-MPC implants while preserving their critical optical, mechanical, and chemical properties. Most importantly, the full regeneration-enabling functionality of the implants was preserved. A full in-depth validation study of e-beam sterilization as a terminal sterilization technique for RHCIII-MPC implants prior to clinical use is therefore merited.

## Data Availability

The datasets presented in this study can be found in online repositories. The names of the repository/repositories and accession number(s) can be found below: The numerical datasets generated for this study can be found in the Figshare Repository DOI:10.6084/m9.figshare.19210326. Image datasets are available upon request from the corresponding author. This study has been previously published as a conference abstract (DOI:10.21037/aes.2018.AB085).

## References

[B1] Altmann S. Emanuel A. Toomey M. McIntyre K. Covert J. Dubielzig R. R. (2010). A Quantitative Rabbit Model of Vaccinia Keratitis. Invest. Ophthalmol. Vis. Sci. 51, 4531–4540. 10.1167/IOVS.09-5106 20375331 PMC2941171

[B3] Chae J. Choi J. Stark W. Elisseeff J. (2013). Extracellular Matrix Characterization of the Acellular Gamma-Irradiated Cornea. Invest. Ophthalmol. Vis. Sci. 54, 5257. Poster Presented at the Association for Research in Vision and Ophthalmology Annual Meeting May 5-9, Seattle WashingtonAvailable at: https://iovs.arvojournals.org/article.aspx?articleid=2150200 (Accessed September 30, 2021).

[B4] CorneaGen VisionGraft® THE CLEAR CHOICE® (2021). CorneaGen VisionGraft® the Clear Choice®. Available at: https://corneagen.com/wp-content/uploads/2021/01/VG100-Cornea_2-Page_2020-01.pdf (Accessed September 30, 2021).

[B5] Dai Z. Ronholm J. Tian Y. Sethi B. Cao X. (2016). Sterilization Techniques for Biodegradable Scaffolds in Tissue Engineering Applications. J. Tissue Eng. 7, 204173141664881. 10.1177/2041731416648810 PMC4874054 27247758

[B6] Daoud Y. J. Smith R. Smith T. Akpek E. K. Ward D. E. Stark W. J. (2011). The Intraoperative Impression and Postoperative Outcomes of Gamma-Irradiated Corneas in Corneal and Glaucoma Patch Surgery. Cornea 30, 1387–1391. 10.1097/ICO.0B013E31821C9C09 21993467

[B7] Dearth C. L. Keane T. J. Carruthers C. A. Reing J. E. Huleihel L. Ranallo C. A. (2016). The Effect of Terminal Sterilization on the Material Properties and *In Vivo* Remodeling of a Porcine Dermal Biologic Scaffold. Acta Biomater. 33, 78–87. 10.1016/J.ACTBIO.2016.01.038 26826528

[B8] European Directorate for the Quality of Medicines & HealthCare (2014). 2.6.14. Bacterial Endotoxins. Eur. Pharmacopoeia 8.0, 161–168.

[B9] Fagerholm P. Lagali N. S. Merrett K. Jackson W. B. Munger R. Liu Y. (2010). A Biosynthetic Alternative to Human Donor Tissue for Inducing Corneal Regeneration: 24-Month Follow-Up of a Phase 1 Clinical Study. Sci. Transl. Med. 2. 46ra61. 10.1126/scitranslmed.3001022 20739681

[B10] Fagerholm P. Lagali N. S. Ong J. A. Merrett K. Jackson W. B. Polarek J. W. (2014). Stable Corneal Regeneration Four Years after Implantation of a Cell-free Recombinant Human Collagen Scaffold. Biomaterials 35, 2420–2427. 10.1016/j.biomaterials.2013.11.079 24374070

[B11] Grimes M. Pembroke J. T. McGloughlin T. (2005). The Effect of Choice of Sterilisation Method on the Biocompatibility and Biodegradability of SIS (Small Intestinal Submucosa). Biomed. Mater Eng. 15, 65–71. 15623931

[B12] Hackett J. M. Lagali N. Merrett K. Edelhauser H. Sun Y. Gan L. (2011). Biosynthetic Corneal Implants for Replacement of Pathologic Corneal Tissue: Performance in a Controlled Rabbit Alkali Burn Model. Invest. Ophthalmol. Vis. Sci. 52, 651–657. 10.1167/iovs.10-5224 20847116

[B13] Herbert A. Edwards J. H. Jones G. L. Ingham E. Fisher J. (2017). The Effects of Irradiation Dose and Storage Time Following Treatment on the Viscoelastic Properties of Decellularised Porcine Super Flexor Tendon. J. Biomechanics 57, 157–160. 10.1016/J.JBIOMECH.2017.04.005 PMC5754329 28449861

[B14] Hoburg A. Keshlaf S. Schmidt T. Smith M. Gohs U. Perka C. (2011). Fractionation of High-Dose Electron Beam Irradiation of BPTB Grafts Provides Significantly Improved Viscoelastic and Structural Properties Compared to Standard Gamma Irradiation. Knee Surg. Sports Traumatol. Arthrosc. 19, 1955–1961. 10.1007/s00167-011-1518-9 21541710

[B15] Hoburg A. T. Keshlaf S. Schmidt T. Smith M. Gohs U. Perka C. (2010). Effect of Electron Beam Irradiation on Biomechanical Properties of Patellar Tendon Allografts in Anterior Cruciate Ligament Reconstruction. Am. J. Sports Med. 38, 1134–1140. 10.1177/0363546509361161 20360605

[B16] International Standards Organization (2012). Sterilization of health care products -- Radiation -- Part 2: Establishing the sterilization dose. ISO 11137-2:2012.

[B17] Islam M. M. Griffith M. Merrett K. (2013). Fabrication of a Human Recombinant Collagen-Based Corneal Substitute Using Carbodiimide Chemistry. Methods Mol. Biol. 1014, 157–164. 10.1007/978-1-62703-432-6_10 23690011

[B33] Islam M. M. Buznyk O. Reddy J. C. Pasyechnikova N. Alarcon E. I. Hayes S. (2018). Biomaterials-Enabled Cornea Regeneration in Patients at High Risk for Rejection of Donor Tissue Transplantation. NPJ Regen Med. 3, 2. 10.1038/s41536-017-0038-8 29423280 PMC5792605

[B18] Kajii F. Iwai A. Tanaka H. Matsui K. Kawai T. Kamakura S. (2018). Influence of Electron Beam Irradiation Doses on Bone Regeneration by Octacalcium Phosphate Collagen Composites. J. Tissue Eng. Regen. Med. 12, e1186–e1194. 10.1002/TERM.2505 28633197

[B19] Kakinoki S. Sakai Y. Takemura T. Hanagata N. Fujisato T. Ishihara K. (2014). Gene chip/PCR-Array Analysis of Tissue Response to 2-methacryloyloxyethyl Phosphorylcholine (MPC) Polymer Surfaces in a Mouse Subcutaneous Transplantation System. J. Biomater. Sci. Polym. Ed. 25. 1658–1672. 10.1080/09205063.2014.939917 25075735

[B20] Lagali N. S. Griffith M. Shinozaki N. Fagerholm P. Munger R. (2007). Innervation of Tissue-Engineered Corneal Implants in a Porcine Model: A 1-year *In Vivo* Confocal Microscopy Study. Invest. Ophthalmol. Vis. Sci. 48, 3537–3544. 10.1167/iovs.06-1483 17652721

[B21] Liu W. Merrett K. Griffith M. Fagerholm P. Dravida S. Heyne B. (2008). Recombinant Human Collagen for Tissue Engineered Corneal Substitutes. Biomaterials 29, 1147–1158. 10.1016/j.biomaterials.2007.11.011 18076983

[B22] Mattern R.-H. Pierschbacher M. D. Cahn F. Tschopp J. F. Malaney T. I. (2001). Collagen/glycosaminoglycan Matrix Stable to Sterilizing by Electron Beam Radiation. Available at: https://patents.google.com/patent/US6969523B1/en (Accessed July 26, 2021).

[B23] Mendes G. C. C. Brandão T. R. S. Silva C. L. M. (2007). Ethylene Oxide Sterilization of Medical Devices: a Review. Am. J. Infect. Control 35, 574–581. 10.1016/j.ajic.2006.10.014 17980234

[B24] Mirazul Islam M. Cėpla V. He C. Edin J. Rakickas T. Kobuch K. (2015). Functional Fabrication of Recombinant Human Collagen-Phosphorylcholine Hydrogels for Regenerative Medicine Applications. Acta Biomater. 12, 70–80. 10.1016/j.actbio.2014.10.035 25448347

[B25] Monaco G. Cholas R. Salvatore L. Madaghiele M. Sannino A. (2017). Sterilization of Collagen Scaffolds Designed for Peripheral Nerve Regeneration: Effect on Microstructure, Degradation and Cellular Colonization. Mater. Sci. Eng. C 71, 335–344. 10.1016/J.MSEC.2016.10.030 27987715

[B26] Noah E. M. Chen J. Jiao X. Heschel I. Pallua N. (2002). Impact of Sterilization on the Porous Design and Cell Behavior in Collagen Sponges Prepared for Tissue Engineering. Biomaterials 23, 2855–2861. 10.1016/S0142-9612(01)00412-4 12069325

[B27] Proffen B. L. Perrone G. S. Fleming B. C. Sieker J. T. Kramer J. Hawes M. L. (2015). Electron Beam Sterilization Does Not Have a Detrimental Effect on the Ability of Extracellular Matrix Scaffolds to Support *In Vivo* Ligament Healing. J. Orthop. Res. 33, 1015–1023. 10.1002/JOR.22855 25676876 PMC4517185

[B28] Seto A. Gatt C. J. Dunn M. G. (2008). Radioprotection of Tendon Tissue via Crosslinking and Free Radical Scavenging. Clin. Orthop. Relat. Res. 466, 1788–1795. 10.1007/S11999-008-0301-9 18512113 PMC2584246

[B29] Shetty R. Rajiv Kumar N. Pahuja N. Deshmukh R. Vunnava K. Abilash V. G. (2018). Outcomes of Corneal Cross-Linking Correlate with Cone-specific Lysyl Oxidase Expression in Patients with Keratoconus. Cornea 37, 369–374. 10.1097/ICO.0000000000001478 29215396

[B30] Stoppel W. L. White J. C. Horava S. D. Henry A. C. Roberts S. C. Bhatia S. R. (2014). Terminal Sterilization of Alginate Hydrogels: Efficacy and Impact on Mechanical Properties. J. Biomed. Mat. Res. 102, 877–884. 10.1002/JBM.B.33070 PMC8218599 24259507

[B31] U.S. Department of Health and Human Services Food and Drug Administration (2012). Guidance for IndustryPyrogen and Endotoxins Testing:Questions and Answers. 240–276. Available at: http://www.fda.gov/Drugs/GuidanceComplianceRegulatoryInformation/Guidances/default.htmhttp://www.fda.gov/BiologicsBloodVaccines/GuidanceComplianceRegulatoryInformation/Guidances/default.htm (Accessed September 21, 2021).

[B32] Zhang J. Davis T. A. Matthews M. A. Drews M. J. LaBerge M. An Y. H. (2006). Sterilization Using High-Pressure Carbon Dioxide. J. Supercrit. Fluids 38, 354–372. 10.1016/J.SUPFLU.2005.05.005

