# Supplementary material for: Electron-Beam Irradiated Recombinant Human Collagen-Phosphorylcholine Corneal Implants Retain Pro-Regeneration Capacity
Source: Front Bioeng Biotechnol. 2022 Jun 13;10:883977. doi: 10.3389/fbioe.2022.883977 (PMC9234199; doi:10.3389/fbioe.2022.883977)
Supplement: Supplementary file 1 [file DataSheet1.docx]

Supplementary Material

# Supplementary Figures and Tables

## Supplementary Figures


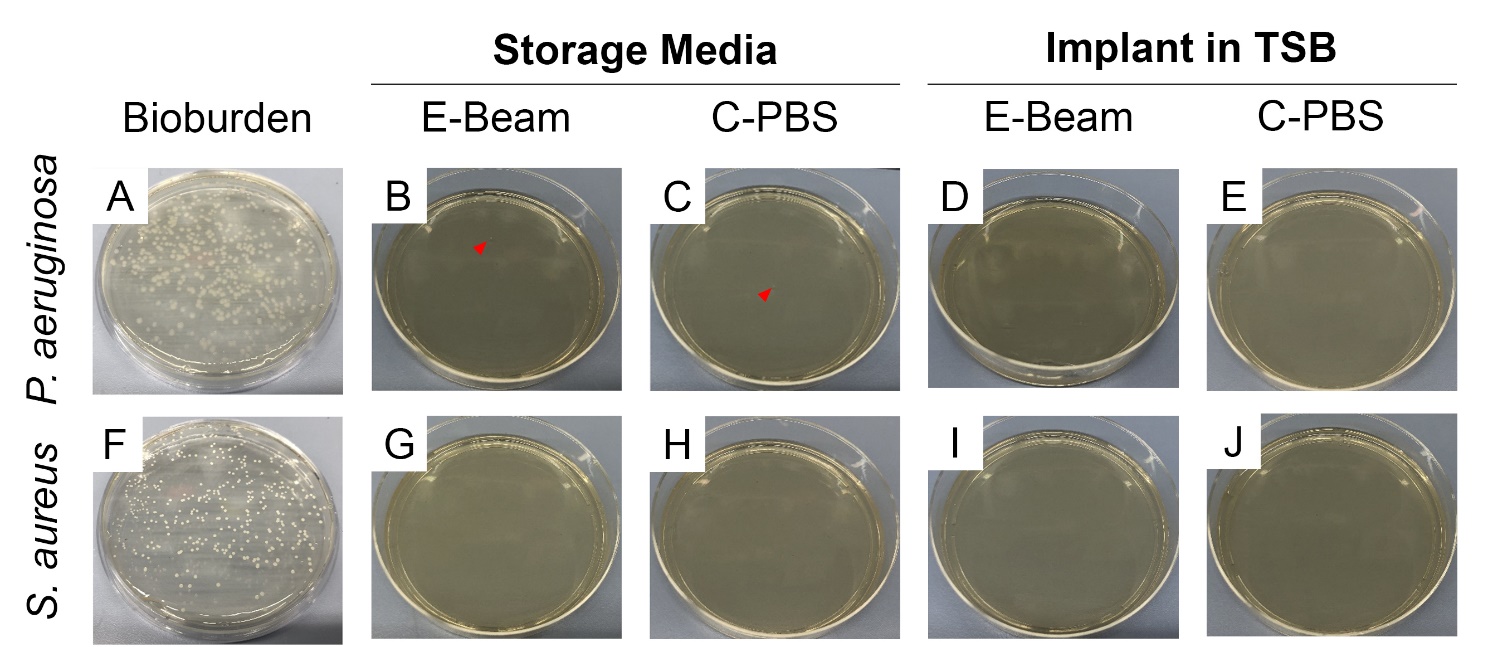


**Supplementary Figure 1.** Bioburden reduction study of RHCIII-MPC implants irradiated at 17 kGy (n=6) or stored in C-PBS (n=6). (A, F) Initial bioburden of *P. aeruginosa* and *S. aureus* used to spike the implants**.** (B, C) Storage media of implants spiked with *P. aeruginosa* (17 kGy n=3, C-PBS n=3) resulted in the persistence of a single CFU. (D, E) No growth was observed from the implants cultured in TSB. (G-J) No colonies of *S. aureus* persisted after the two methods of sterilization (17 kGy n=3, C-PBS n=3).

## Supplementary Tables

**Supplementary Table 1.** Simple linear regression of corneal epithelial cell proliferation

|  | **EB** | **C-PBS** | **TC** |
| --- | --- | --- | --- |
| **Best-fit values** |  |  |  |
| Slope | 18.57 | 33.68 | 22.17 |
| Y-intercept | -97.67 | -545.5 | 1.375 |
| X-intercept | 5.26 | 16.2 | -0.06203 |
| 1/slope | 0.05385 | 0.02969 | 0.04511 |
| **Std. Error** |  |  |  |
| Slope | 3.983 | 2.748 | 3.124 |
| Y-intercept | 206.5 | 143.7 | 163.4 |
| **95% Confidence Intervals** |  |  |  |
| Slope | 9.152 to 27.99 | 26.96 to 40.40 | 14.52 to 29.81 |
| Y-intercept | -585.9 to 390.6 | -897.3 to -193.8 | -398.5 to 401.3 |
| X-intercept | -40.92 to 21.84 | 7.025 to 22.73 | -26.86 to 13.75 |
| **Goodness of Fit** |  |  |  |
| R squared | 0.7564 | 0.9616 | 0.8935 |
| Sy.x | 234.1 | 161.5 | 183.7 |
| **Is slope significantly non-zero?** | |  |  |
| F | 21.74 | 150.2 | 50.34 |
| DFn, DFd | 1, 7 | 1, 6 | 1, 6 |
| P value | 0.0023 | <0.0001 | 0.0004 |
| Deviation from zero? | Significant | Significant | Significant |
| **Equation** | Y = 18.57*X - 97.67 | Y = 33.68*X - 545.5 | Y = 22.17*X + 1.375 |
